# Annotations

**Published:** 1909-05-22

**Authors:** 


### ANNOTATIONS.

### School Clinics.

Those acute observers who predicted that the medical inspection of school children was but the point of a wedge whose ultimate effect must be free treatment of the pathological conditions found, seem likely to be speedily justified. The problems of racial degeneration, of the relations of physical ill-health and of mental and moral deterioration, of heredity, crime, alcoholism, and many other detrimental conditions, are much too large to be discussed otherwise than at great length. But it is interesting to observe that the recommendations of the Education Committee of the London County Council in the matter of subsidies to hospitals and dispensaries do not by any means command unanimous support on that committee. Indeed, the majority of a special sub-committee appointed to consider the matter were in favour of the formation of school clinics. The dilemma with which public bodies having the responsibility of looking after the youthful proletariat are faced can be put almost in a nutshell. Owing partly to the ignorance, uncleanly and improvident habits, carelessness, overcrowding, poverty, bad feeding, and many other factors of the life of the poor (in towns especially), their children suffer from a large variety of entirely preventable causes of ill-health. While prophylaxis is what must eventually be aimed at, the treatment of that which now exists must also be undertaken if the nation's most valuable asset, the efficiency of its governing class, is not to be seriously impaired. This treatment, as, for instance, of dental caries, otorrhœa, errors of refraction, and many others of the commoner lesions, demands skilled medical attention of a kind which the parents unaided are nearly always unable to provide. The alternatives are: to extend the present hospital and dispensary system by subsidies, to start school clinics, and to do nothing. The last is out of the question, the second is very expensive, the first is regarded by the British Medical Association as an extension of a system of sweating already unbearable. Moreover, the majority of the special committee believe that the expense of any workable system of subsidies would be greater than that of the establishment of clinics, and that the practical efficiency of the scheme would be less.

### The Stage and Medical Charity.

A few months ago we recalled the fact that both Medicine and the Drama may claim Sir Charles Wyndham as a member of their ranks. We did not then say—as was well known to us and many others, that this distinguished actor has always retained a practical interest in his former profession, and has often aided in the furtherance of medical charities. This friendly co-operation and sympathy between the stage and the hospitals is again brought prominently before our notice by the publication in the *Times* of May 18 of recent correspondence between the Hon. W. F. D. Smith, chairman of King's College Hospital, and Sir Charles Wyndham, President of the Actors' Benevolent Fund. The former in his letter explains that for some time past the committee of management have wished to acknowledge suitably their deep obligation to the theatrical profession for the unvarying kindness with which its members have freely given their services on many occasions on behalf of the hospital. Realising that they cannot adequately express their gratitude to individuals, they have decided, as a permanent token of recognition of the friendly help which they have received, to invite the committee of the Actors' Benevolent Fund to name a bed in the New King's College Hospital now being erected on Denmark Hill, and also to nominate a member of the theatrical profession for election as an honorary life governor of the hospital. Sir Charles Wyndham's reply expresses warm appreciation of the honour proposed to be conferred on the Fund by the Hospital, and of the generous and graceful terms in which it was conveyed. "Whatever services the stage has been privileged to render to medicine are no more than a just tribute to the unstinted beneficence invariably extended by your profession to theirs." The bed in the new hospital will most appropriately be christened in the name of Henry Irving; while the committee of the Actors' Benevolent Fund has nominated Sir Charles Wyndham as an honorary life governor of the hospital. Both decisions cannot fail to receive general approval from the members of both professions, and of the hospital with whom the idea originated.

### St. Bartholomew's and the City.

Last week we gave a short account of the recent Ceremony at St. Bartholomew's Hospital, when the Lord Mayor opened the New Pathological Block of that institution. The new buildings are strikingly well adapted for the routine hospital investigations, for the instruction of the clerks and dressers in pathological methods, and for higher research into the problems of disease. The Lord Mayor made sympathetic reference to the unbroken relations, through nearly eight centuries, between the City of London and its great General Hospital—the only one within the City boundaries. While showing clear appreciation of the worldwide benefits which will issue through the work of this new and elaborate centre of medical research, the Lord Mayor also spoke with knowledge and feeling of the grave financial embarrassment which now threatens the hospital. Unless active and generous help is soon forthcoming this great and ancient institution will be crippled to an extent scarcely yet comprehensible to those who have always thought of it as a monument of wealth and stability. Following the Lord Mayor's speech and the ominous announcements in the first annual report of Lord Sandhurst, the new treasurer of St. Bartholomew's, the former called a meeting on May 14, at the Mansion House, of the Masters and Wardens of the leading City Companies, to confer with them upon the financial crisis which has befallen their hospital. A letter was then read assuring the Lord Mayor that the Prince of Wales, as President of the Hospital, is alive to its urgent needs, and earnestly hopes that the City will see to it that the work of its one general hospital is not curtailed for the first time by lack of income. We propose to consider this grave state of affairs in an early number of The Hospital.

